# The "Meat" Treatment of Sprue

**Published:** 1910-03-05

**Authors:** 


### THE "MEAT" TREATMENT OF SPRUE.

Cantlie holds that the "meat" treatment of sprue will not fail if it is carried out early and systematically. It consists essentially of the following points:—

The patient is put to bed, at any rate for a few days. A wet pack is applied—a towel, or double-layer of flannel, large enough to reach from nipples to groins, and sufficiently wide to reach half-way round either loin—is wrung out in warm water and laid upon the abdomen. Round the body a large bath-towel is wrapped, pulled tight and fixed by safety-pins. The wet pack is kept on for two hours at a time, and is to be applied morning and evening. The patient, unless very ill, is given five ounces of pounded beef, lightly cooked, for breakfast, luncheon and dinner, with salt to taste, unless the mouth soreness prohibits its use. Every two hours the patient is given jellied beef-tea, beef-jelly, calf's-foot-jelly, or plain jelly, all home made; the jelly is given also during the night should the patient wake. One and a half drachms of castor-oil are given every morning for the first three mornings, and 3 grains of santonin morning and evening for three days. The effect of this treatment is that the stools at once become "bilious"; the bile, which seems to have been pent up, owing to previous milk treatment, flows with great freedom when meat is given, and at first the stools are copious, loose, and as many as two or three daily. By the third day, in all probability, no stool is passed, and when on the fourth day the motion is seen it is not infrequently fairly solid, fæcal and deeply bile-stained.

The diet may now (the fourth day) be increased, a poached egg is added to the beef at breakfast and dinner; pounded chicken may be given at the midday meal instead of beef. On the sixth day the meat or chicken may be finely minced—thrice passed through the mincing machine—in place of being pounded. By the eighth day the patient may have a cut off a joint, the undercut of a piece of roast beef being the best.

As soon as the stools are solid, or fairly solid, vegetables may be added to the diet—stewed celery, stewed sea-kale, or vegetable marrow, also pulled and baked bread—thin slices of bread kept in a hot oven for 20 minutes until dry and crisp. The patient's diet is now fairly varied, and additional food can be tried, as judgment directs. For beverages, either rice-tea or china-tea are permissible. Rice-tea is made by roasting some rice in the oven until brown; then placing two tablespoonfuls of it in a jug and pouring over it one pint of boiling water and allowing the decoction to stand for 15 minutes, when the rice is strained off and the "tea" used as a drink. China tea is made by taking the finest procurable, placing half a teaspoonful in a strainer over a breakfast cup and pouring boiling water over the leaves. Either of these teas, or each used alternately, may be given 15 minutes after finishing food—never with the meat, of course.

